# Supplementary figures and images for: Genome-Wide Transcription During Early Wheat Meiosis Is Independent of Synapsis, Ploidy Level, and the Ph1 Locus
Source: Front Plant Sci. 2018 Dec 4;9:1791. doi: 10.3389/fpls.2018.01791 (PMC6288783; doi:10.3389/fpls.2018.01791)

**A**

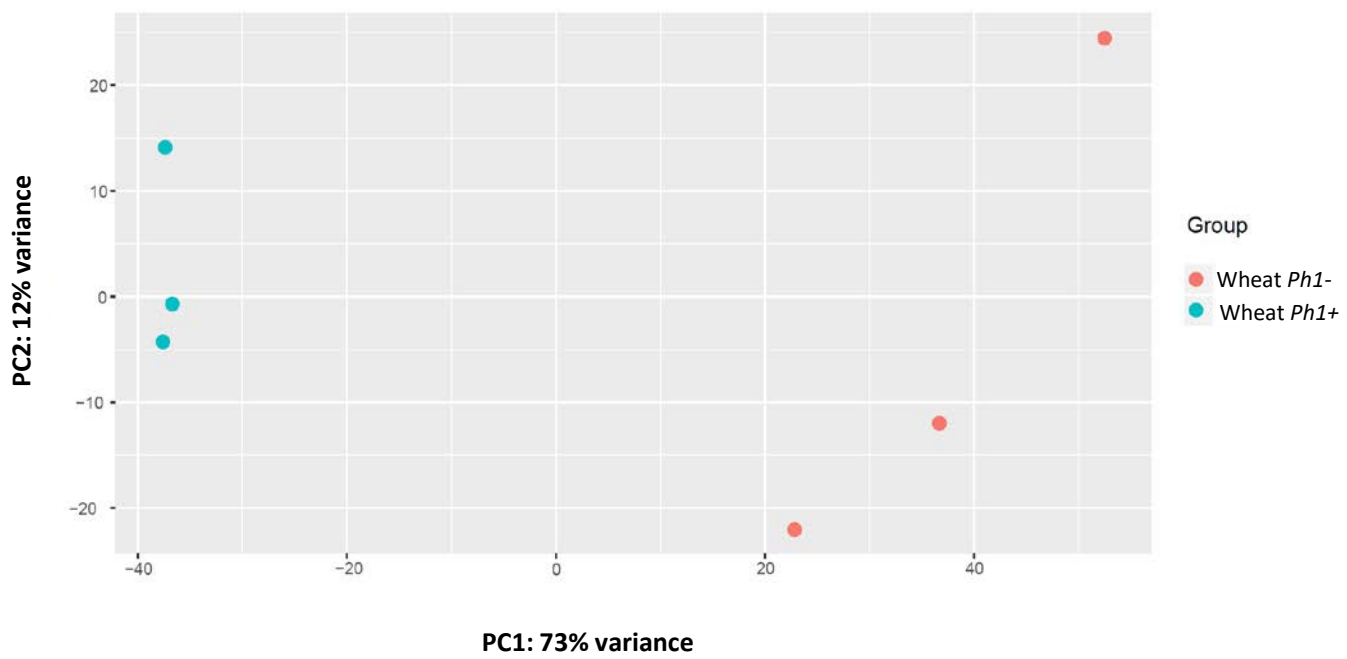

**B**

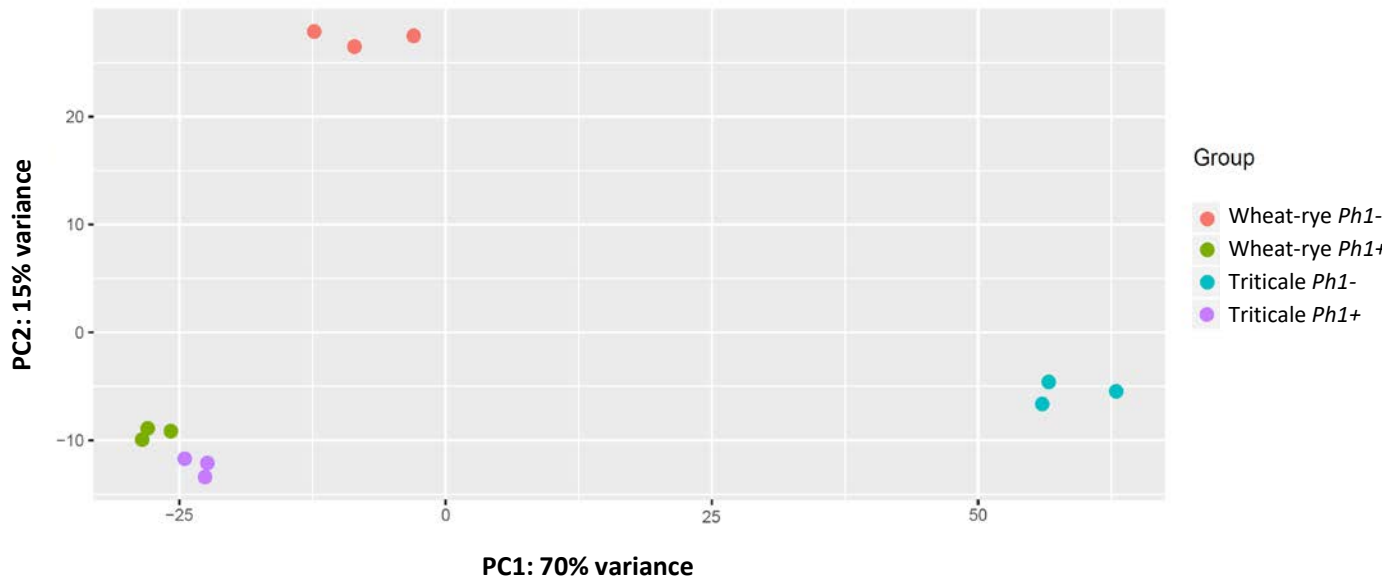

Supplement: FIGURE S1 — Principal component analysis (PCA) of samples analyzed in this study. Three biological replicates were produced per genotype. (A) PCA for the six wheat samples, three containing the Ph1 locus (Ph1+) and three lacking it (Ph1-). The x and y axis represent the two principal components of the total variance, 73 and 12%, respectively. (B) PCA for 12 wheat–rye hybrid and triticale samples. Three hybrids containing and three lacking Ph1, and three triticale containing and three lacking Ph1. The x and y axis represent the two principal components of the total variance, 70 and 15%, respectively. [file Data_Sheet_1.PDF]

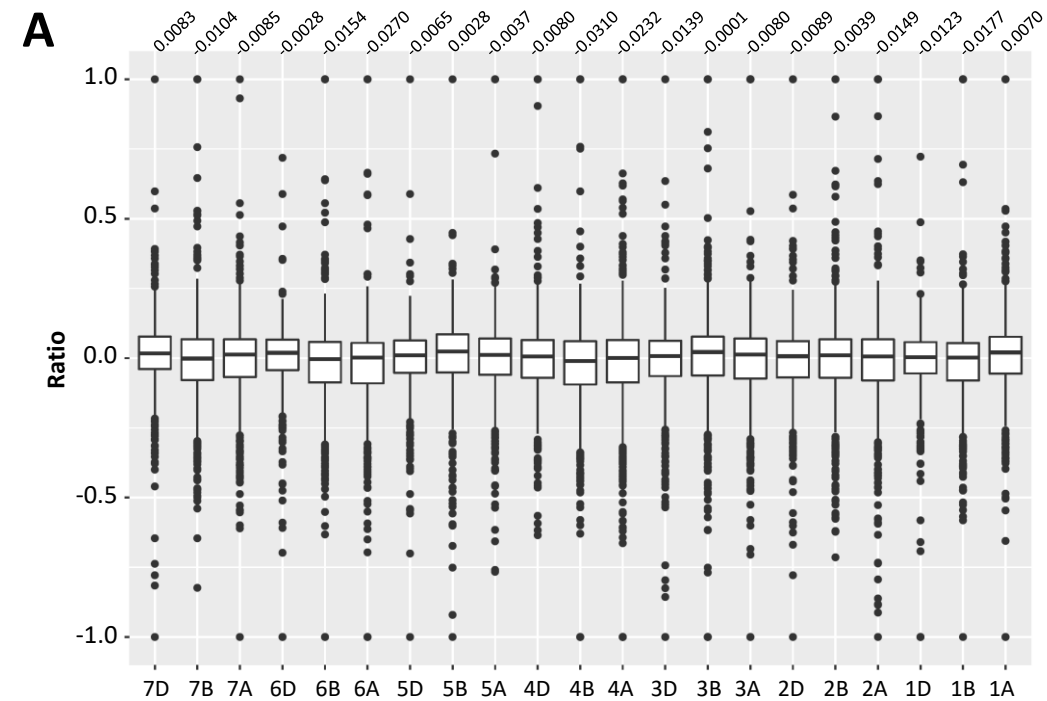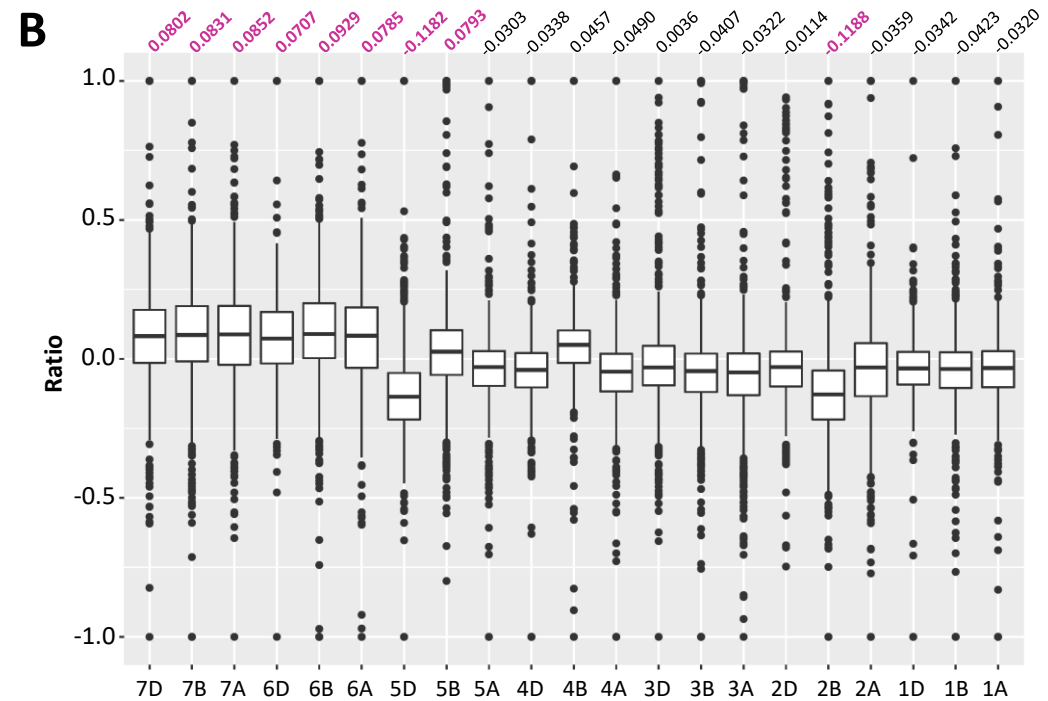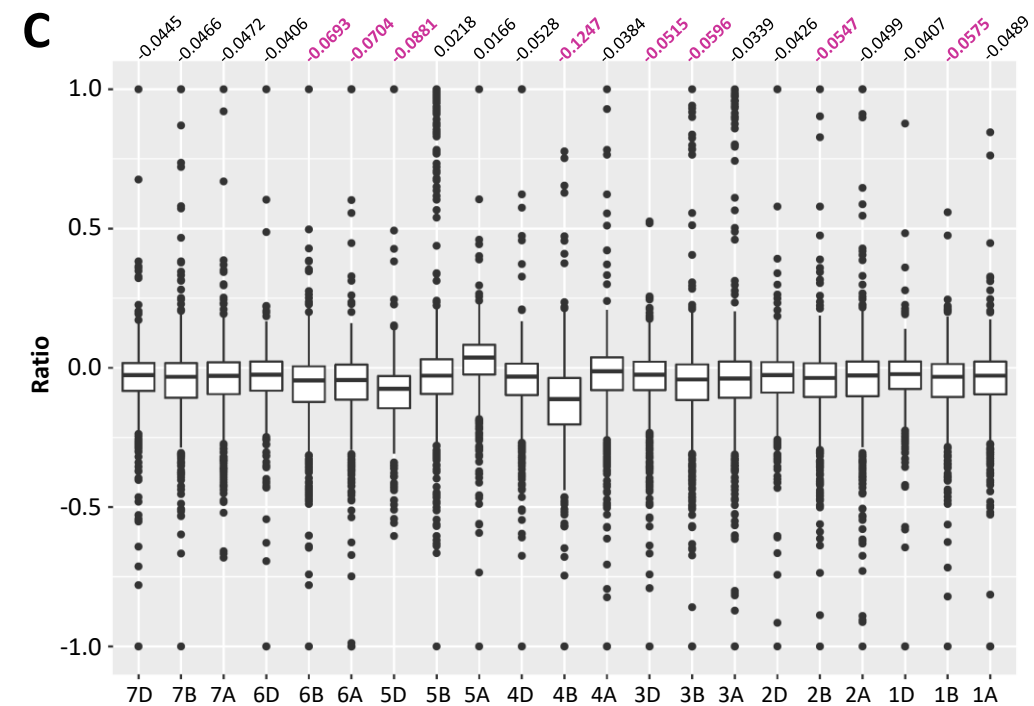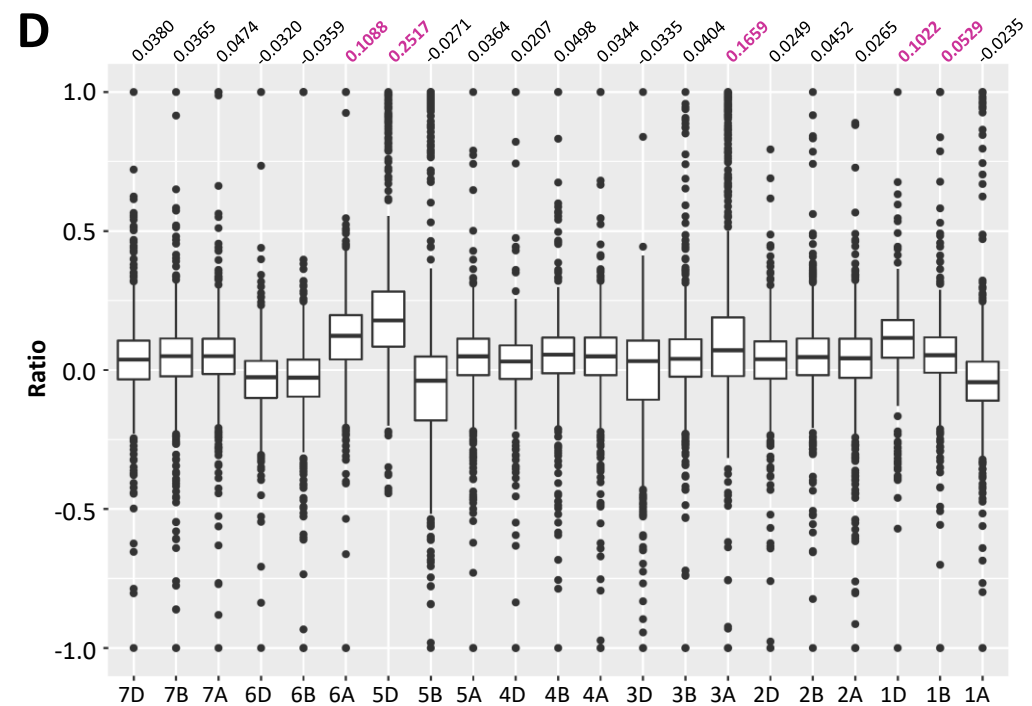

Supplement: FIGURE S2 — Representation of the ratio of coverage along all chromosomes using Box plots. (A) Box plot comparing wheat–rye hybrids and triticale, both containing the Ph1 locus. (B) Box plot comparing wheat in the presence and absence of Ph1. (C) Box plot comparing wheat–rye in the presence and absence of Ph1. (D) Box plot comparing triticale in the presence and absence of Ph1. Arithmetic mean values of the coverage ratio per chromosome are indicated on the upper part of the plots. Mean values >0.05 and <-0.05 are highlighted in magenta. [file Data_Sheet_2.PDF]

Wheat *Ph1+*  
vs.  
*wheat Ph1-* sample 1

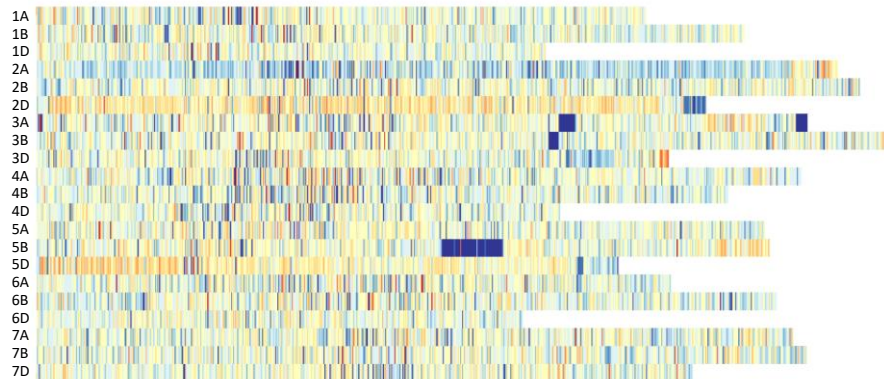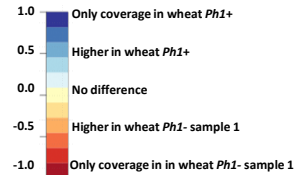

Wheat *Ph1+*  
vs.  
*wheat Ph1-* sample 2

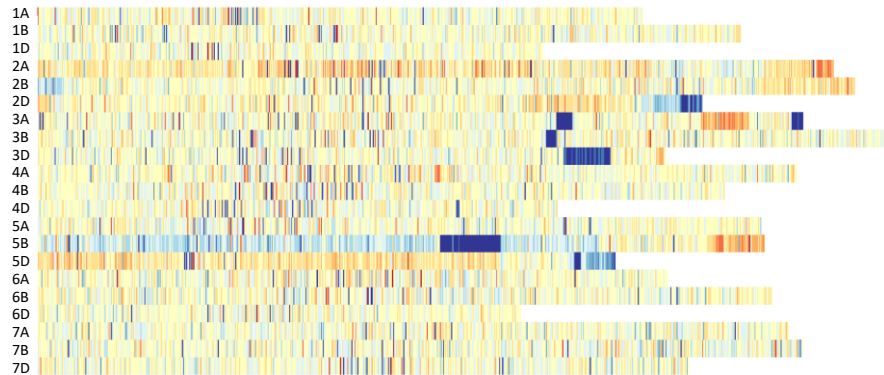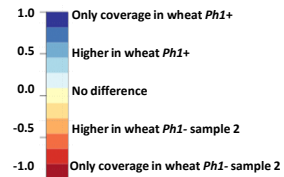

Wheat *Ph1+*  
vs.  
*wheat Ph1-* sample 3

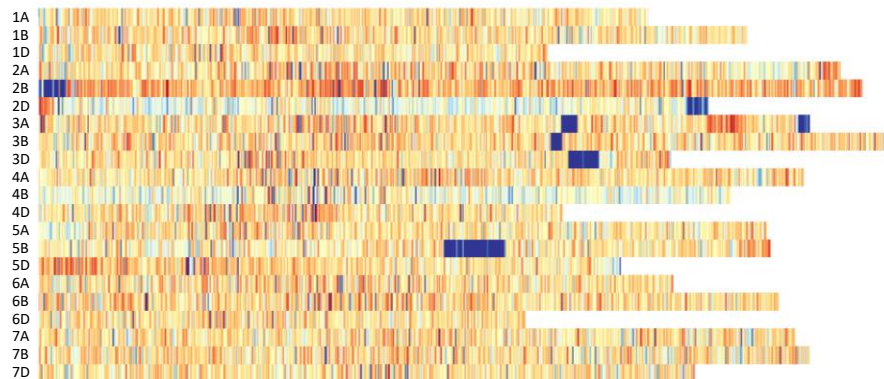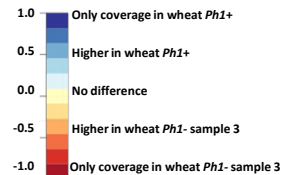

Supplement: FIGURE S3 — Chromosome coverage plots of wheat containing Ph1 (Ph1+) (three samples pooled together) vs. each individual sample of wheat lacking Ph1 (Ph1-). Heatmaps show that each wheat sample lacking Ph1 is different. Several deletions (visualized in dark blue) are common to all three samples, but other deletions and chromosomes rearrangements are different between them. [file Data_Sheet_3.PDF]

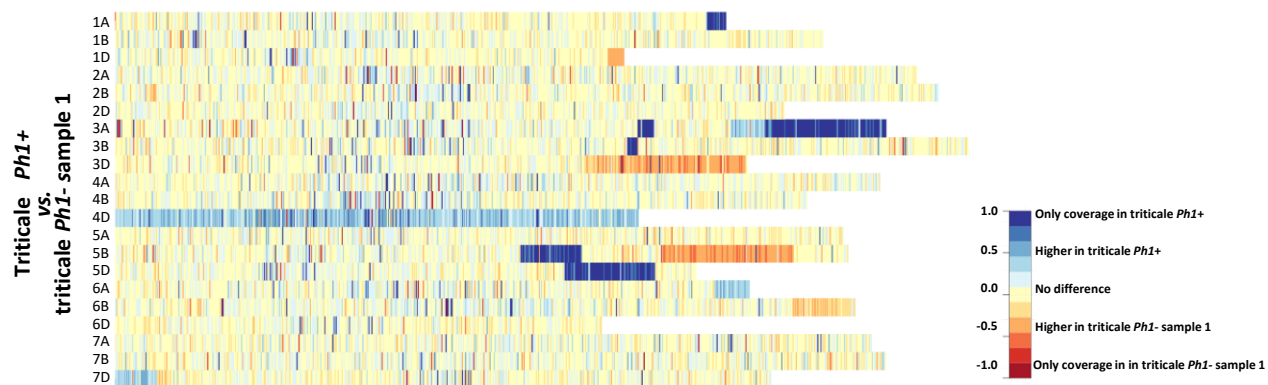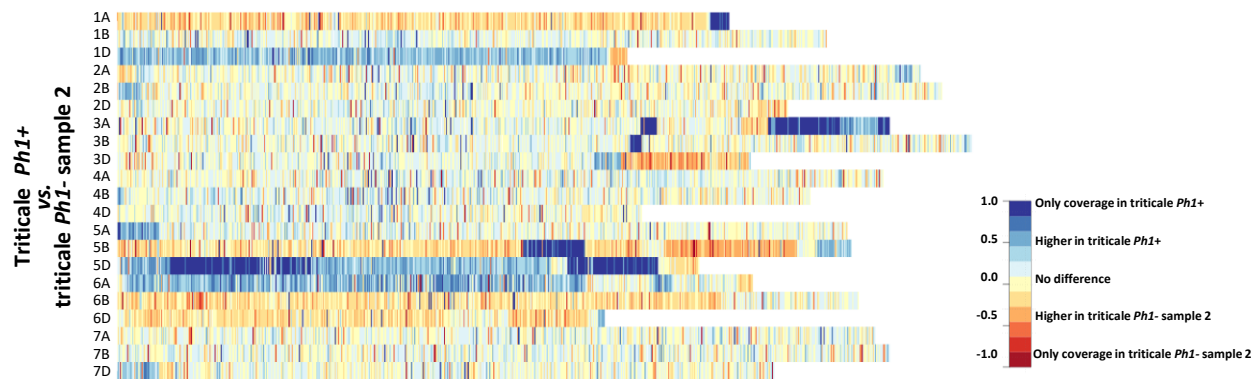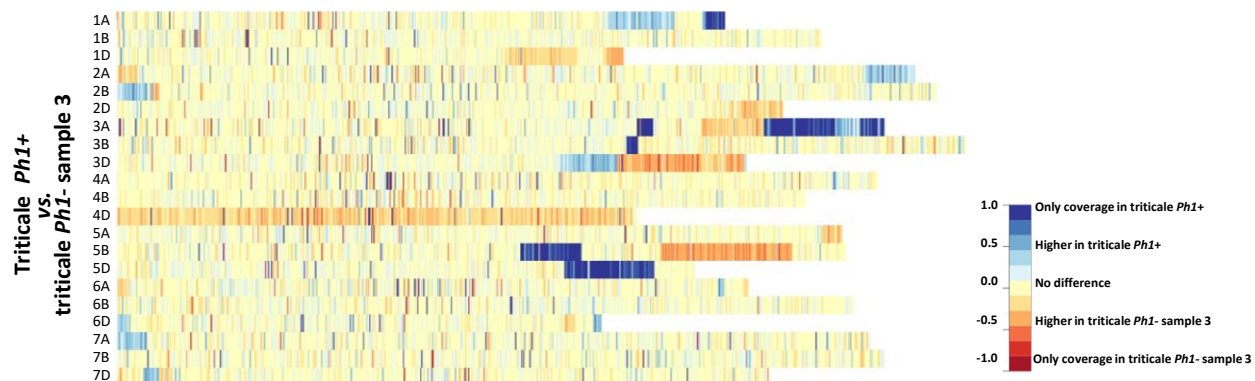

Supplement: FIGURE S4 — Chromosome coverage plots of triticale containing Ph1 (Ph1+) (three samples pooled together) vs. each individual sample of triticale lacking Ph1 (Ph1-). Heatmaps show that each triticale sample lacking Ph1 is different. Several deletions (visualized in dark blue) are common to all three samples, but other deletions and chromosomes rearrangements are different between them. [file Data_Sheet_4.PDF]

**A**Triticale *Ph1+*Triticale *Ph1-*

20 cm

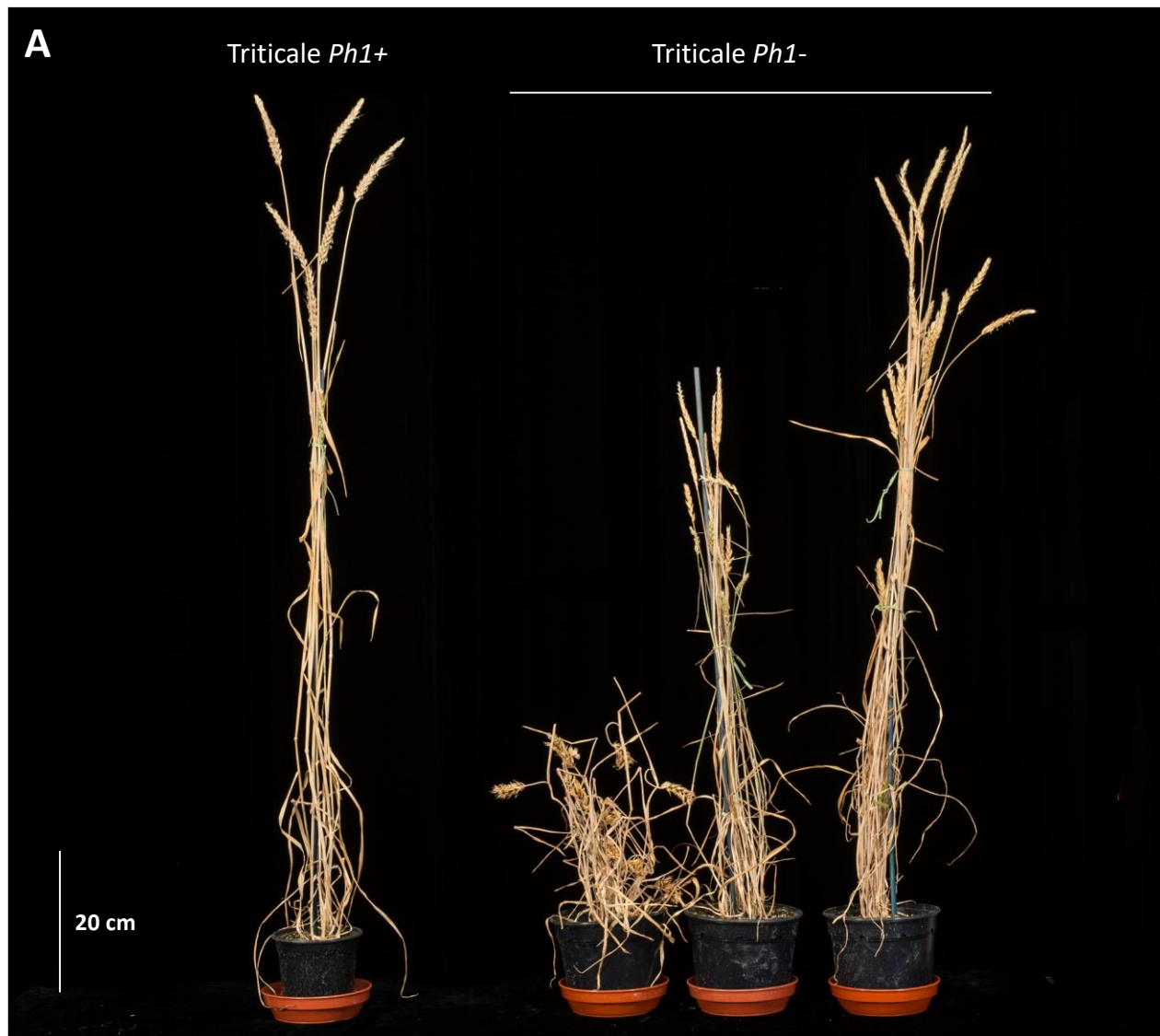**B**Triticale *Ph1+*Triticale *Ph1-*

5 cm

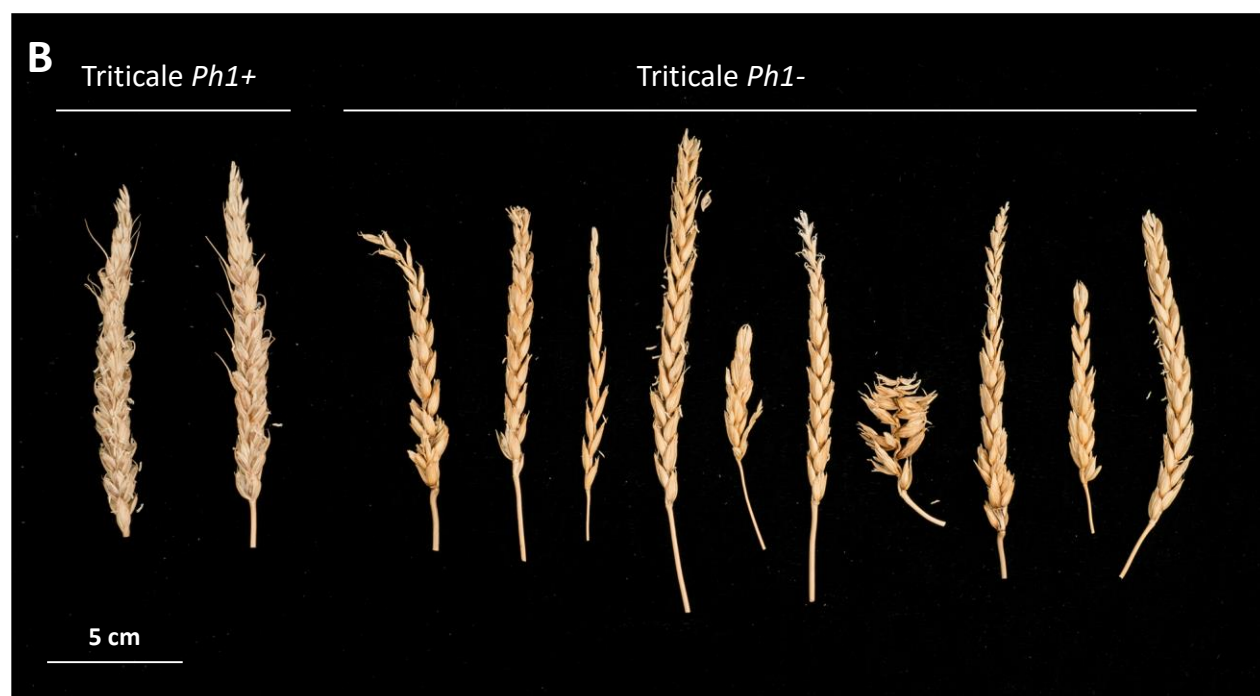

Supplement: FIGURE S5 — Morphology of whole plants (A) and spikes (B) of triticale containing the Ph1 locus (Ph1+) and lacking it (Ph1-). Plant and spike morphology of all triticale containing Ph1 was perfectly normal; however, every triticale lacking Ph1, was morphologically different, some exhibiting very abnormal phenotypes. [file Data_Sheet_5.PDF]
